# Chemical Difference between Living and Dead Protoplasm

**Published:** 1882-04

**Authors:** 


### A CHEMICAL DIFFERENCE BETWEEN LIVING AND DEAD PROTOPLASM.

From various experiments (chiefly with protoplasm of plants, also with infusoria), Herren Loew and Bokorny find (*Pflüger's Archiv*) that living protoplasm possesses in an eminent degree the property of reducing the noble metals from solutions, and that this property is lost when death occurs. "It may well be inferred," say the authors, "that the mysterious phenomenon denoted by the name of 'Life' depends essentially on these reducing atom-groups. In the present state of science we explain these 'groups in motion,' these springs of life phenomena, as aldehyde groups, but would by no means exclude some different and better mode of explanation."—*British Medical Journal, Lancet and Clinic.*

